# Accelerating Tomato Breeding by Exploiting Genomic Selection Approaches

**DOI:** 10.3390/plants9091236

**Published:** 2020-09-18

**Authors:** Elisa Cappetta, Giuseppe Andolfo, Antonio Di Matteo, Amalia Barone, Luigi Frusciante, Maria Raffaella Ercolano

**Affiliations:** Department of Agricultural Sciences, University of Naples Federico II, Via Università 100, 80055 Naples, Italy; elisa.cappetta@unina.it (E.C.); giuseppe.andolfo@unina.it (G.A.); antonio.dimatteo@unina.it (A.D.M.); ambarone@unina.it (A.B.); fruscian@unina.it (L.F.)

**Keywords:** tomato, genetic breeding value, training population, genotyping, marker effect, phenotyping, selection schemes

## Abstract

Genomic selection (GS) is a predictive approach that was built up to increase the rate of genetic gain *per* unit of time and reduce the generation interval by utilizing genome-wide markers in breeding programs. It has emerged as a valuable method for improving complex traits that are controlled by many genes with small effects. GS enables the prediction of the breeding value of candidate genotypes for selection. In this work, we address important issues related to GS and its implementation in the plant context with special emphasis on tomato breeding. Genomic constraints and critical parameters affecting the accuracy of prediction such as the number of markers, statistical model, phenotyping and complexity of trait, training population size and composition should be carefully evaluated. The comparison of GS approaches for facilitating the selection of tomato superior genotypes during breeding programs is also discussed. GS applied to tomato breeding has already been shown to be feasible. We illustrated how GS can improve the rate of gain in elite line selection, and descendent and backcross schemes. The GS schemes have begun to be delineated and computer science can provide support for future selection strategies. A new promising breeding framework is beginning to emerge for optimizing tomato improvement procedures.

## 1. The Tomato Genetic Background

Tomato (*Solanum lycopersicum*) is one of the most important vegetable crops worldwide. It possesses unique properties, offering a rich source of minerals (potassium, magnesium, phosphorus) and antioxidant compounds, which prevents cardiovascular and cancer diseases, and strengthens our immune system [1]. Tomato is an autogamous diploid species, with a modest genome size (~900 Mb) and a relatively short life cycle. As a model plant, numerous genetic and molecular tools have been developed for tomato species, including a high-quality draft genome sequence, high-density genetic maps, high-throughput molecular markers, introgression lines, and mutant collections (Tomato Genome Consortium—[2]). Moreover, hundreds of genomes from landraces, cultivars, and wild relatives have been re-sequenced, revealing a relatively low molecular diversity but a high rate of chromosome rearrangements due to traces of wild introgressions [3].

Tomato genetic basis became narrow along the process of domestication, preventing intra-populational breeding strategies to provide satisfactory genetic gains [4]. Besides the low genetic variability that limits breeding gains of conventional and modern selection schemes, the tomato is tolerant to inbreeding and this allows the generation and maintenance of inbred lines. Therefore, the recombination of the genetic variability represents an excellent alternative for obtaining superior genotypes [4,5]. Moreover, the retaining of genome segments from wild relatives, used to introgress agronomically relevant traits such as resistance to diseases and quality traits, largely contributes to the genetic variability within the cultivated tomato gene pool [6,7,8].

In the early 1980s, the development of different molecular marker systems drastically changed the fate of plant breeding. A high saturated tomato reference linkage map based on *L. esculentum* LA925 (E6203) and *L. pennellii* LA716 interspecific population is available and the growing tomato sequencing projects are providing additional information for developing more resolving genetic markers [9]. However, the lack of enough DNA markers that detect polymorphism within the cultivated species remains a major issue in tomato crop [10]. Thus, most recently significant efforts were attempted to exploit intraspecific high-resolution genetic markers such as Single Nucleotide Polimorphisms (SNPs) and Insertion and Deletion (InDels to detect polymorphism among closely related individuals [11,12].

Molecular markers were mainly integrated into traditional phenotypic selection (PS) by applying marker-assisted selection (MAS) to improve the plant selection process through the inclusion of chromosomal segments containing quantitative trait loci (QTLs) or single genes [13,14,15,16,17]. Several research articles concerning the identification of tomato QTLs and major genes conferring resistance to biotic and environmental stresses have been reviewed in [6,18]. Molecular markers have been also used in tomato to map genes or QTLs for environmental stresses and some flower and fruit-related traits, reviewed in [19]. However, MAS is more suitable for application concerning simple traits with a few major-effect genes [20,21]. Genomic selection (GS), which uses genome-wide markers to predict breeding values, may greatly improve the selection gain in breeding programs for complex traits controlled by several minor genes. In last year, pioneer studies concerning the application of GS to transfer yield-related traits in tomato varieties from wild related species were reported [22,23] as well as to assess the potential of GS to increase soluble solids content and fruit weight in F1 tomato varieties [24] and to develop bacterial spot resistant tomato lines [25]. GS models were widely exploited for predicting phenotypes of progeny and parents, although the efficiency varied depending on the parental cross combinations and the selected traits [26]. Optimized and validated GS protocols are still needed in the tomato. Several GS programs in this species are still in progress, thus the impact of factors affecting the implementation and the accuracy of the model has not yet been evaluated while their optimization for tomato breeding is still required. Among these factors, the genetic structure of species, phenotyping procedures, size of populations, genetic relationship between individuals of population assessed, genotyping platforms, marker quality metrics, and design of GS schemes should be further investigated.

Here, we illustrate the main progress achieved in plant GS and discuss the main challenges of its application in tomato breeding. Tomato GS schemes within and across breeding generations, as well as its potential to select parents based on their assessed GEBV, are also described.

## 2. Potential of GS in Plant

**Figure 1:**
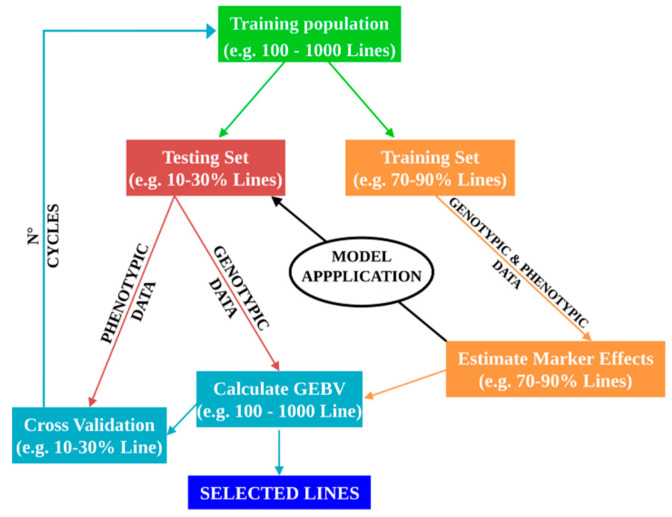
Flowchart of a genomic selection (GS) breeding program. GS overview with cross-validation using a training set (70–90% out of 100–1000 lines) to estimate marker effects in order to obtain a genomic estimated breeding value (GEBV) of lines in the testing set (10–30% out of 100–1000 lines). Finally, phenotypic and genotypic data of the training set are used to set up the prediction model.

Genomic selection (GS) provides new opportunities for increasing the efficiency of plant breeding programs for traits with polygenic inheritance [21,27,28,29,30]. The potential breeding value of an individual is estimated using large scale genomic-based data such as SNPs. Recent high-throughput genotyping (HTG) systems help to generate several thousand of markers allowing entire genomes to be scanned. The allelic association of marker loci with the phenotypes can be used to predict the phenotypic value of a candidate for selection. A genomic estimated breeding value (GEBV), expressed as a linear function of marker effects, is calculated for each breeding candidate. GS combines genotypic and phenotypic data from a training population (TRN) in a training set (TRS) to obtain the GEBVs of a testing set (TST) which has been genotyped but not phenotyped. The GS model will be then employed to predict breeding values of not phenotyped individuals in the next selection step (Figure 1). The first report concerning GS simulation in plants was provided by Meuwissen and colleagues [31]. The authors provided a comparison among linear regression, best linear unbiased prediction, and Bayesian prediction methods for estimating the relationship between true breeding values and estimated breeding values in order to develop suitable GS models in plant.

Genomic screening of breeding populations can accelerate the genetic gain obtained at each cycle, reducing up two-third the time required for selection [21], especially when the selection is performed for traits with low heritability. Although the effect of each marker is very small, a large amount of genome-wide marker information has the potential to explain all the genetic variance [29]. However, the implementation of GS in real plant breeding schemes can be challenging for plant breeders. The number of candidates that can enter a plant breeding program is typically limited by costs and time of the breeding cycle that ultimately impact on the rate of genetic gain in crops. To date, several GS-based breeding strategies have been conceived in different crops including wheat, maize, rice, barley, soybean, tomato (Table 1) increasing rates of genetic gain [32,33,34,35]. The extensive use of GS in plants breeding requires to reduce GS costs, develop cost-effective genotyping, phenotyping platforms, create diverse and updatable TRN, develop highly efficient and multifunctional genomic prediction models, enhance agronomic procedures for shortening breeding cycle time, build up a strong decision support system, and establish open-source breeding programs. In addition, several primary factors that affect GS, including marker density, training-set size, species genetic relationship between TRN and TST, population structure, phenotyping, and statistical model reliability should be carefully calibrated [36,37].

The prediction accuracy of a GS approach in plant breeding programs is strictly dependent on population linkage disequilibrium (LD), which increases with the number of recombination events [38]. GS is most accurate if the training and prediction populations are closely related and share long-range haplotypes [39,40]. Different studies underlined the impact of increasing the relatedness by including more related crosses in TRN rather than increasing the TRN size by adding unrelated or less-related crosses [26,41,42,43]. However, continuously using closely related populations to achieve better prediction would narrow down the genetic basis and ultimately reduce the genetic gain that would be achieved in long-term GS [38,44,45]. Therefore, the TRN–TST relationship should be balanced to preserve the genetic gain for both short-term and long-term selection [46]. It is noteworthy to underlie that the quality of phenotyping strongly affect the accuracy of GS [47]. To date, phenotyping is labor and time-intensive and is also largely affected by human errors and biases. For these reasons, robotic devices and aerial vehicles are becoming a big opportunity to increase the accuracy of the phenotypic estimations, which in turn can be used in statistical models [48,49,50,51,52]. The development of statistical models capable of accurately predict marker effects has led to the breakthrough of GS increasing the rate of genetic gain per unit of time. However, the ability of different models to predict plant performances need to be evaluated. Heffner et al. [53] reported comparable values among Bayes-A, Bayes-B, and RR-BLUP models for 13 traits in 374 wheat lines. Besides, in 413 rice empirical data, small differences in accuracy were found with different statistical models (least absolute shrinkage and selection operator-LASSO, Bayes-B, and RR-BLUP) [54]. In addition, some studies report that the accuracy of prediction change when different statistical models were applied to different traits. For instance, Perez-Rodriguez et al. [55] applied seven different statistical models in wheat on two traits (heading date and grain yield), revealing that Reproducing Kernel Hilbert Spaces (RKHS) had the best accuracy for heading date, whereas Bayes-A and Bayes-B were the best in evaluating grain yield. Given these differences, it is advisable for researchers to carefully train models before the implementation of GS in crop breeding programs.

## 3. Lesson from Other Species

The effectiveness of GEBVs for prediction was mainly demonstrated in polyploid wheat [27,52,53,54,55,56,57] but studies are also available for diploids rice [54,58], barley [59,63], soybean [60,61], maize [27,62] and tomato [22,23,24,25]. Lorenzana and Bernardo [59] obtained GEBV accuracies between 0.64 and 0.83 using 150 DHs (doubled-haploid) barley lines and 223 Restriction Fragment Length Polymorphism (RFLP) markers to improve grain yield and amylase activity. Similarly, Xu [58], in rice, estimated the GEBV accuracy at 0.16 and 0.98 for yield and white sugar, respectively, using 210 inbred lines and 270,082 SNPs. Drought-tolerant maize varieties, referred to as the “*AQUAmax*” hybrids, significantly improving yield stability under water-limitation were generated by GS [64].

Wimmer et al. [54] obtained GEBV accuracies between 0.43 and 0.51 for grain yield using three different statistical models in 254 CIMMYT wheat lines, whereas Perez-Rodriguez and colleagues [55] recorded an accuracy of roughly 0.7 for the same trait but in 306 CIMMYT wheat lines.

These studies clearly indicated that the training population size is an important driver in determining the model accuracy. Indeed, Xu [58], using a TRN made of 210 rice varieties, obtained an accuracy of 0.26 for flowering time, whereas Wimmer et al. [54] performed an accuracy of 0.50 using 413 genotypes for the same trait. High accuracy levels were achieved in the par excellence model species *Arabidopsis thaliana.* In fact, an accuracy of ~0.9 was obtained for flowering time using 415 RIL population [59], whereas lower values (~0.7) were reported when a TRN made of 199 inbred lines was used [54]. Another key component for genomic selection success is the marker density. Generally, the higher the number of markers used, the better the accuracy obtained. A critical example was given in wheat by Heffner et al. [53] and Perez-Rodriguez et al. [55]. The former reported accuracy of 0.21 for grain yield using 374 individuals and 1158 Diversity arrays technology (DArT) markers. By contrast, the latter achieved better results for the same trait with a similar number of samples but higher marker density (accuracy of 0.7 obtained with 1717 DArTs). Generally, high marker density can augment prediction accuracy until the maximum level [65,66,67,68]. In addition, the marker density required for outcrossing species is higher than that for self-pollinated species [68,69] and the marker numbers required for natural populations with higher LD are normally higher than those for biparental populations [69,70]. However, accuracy and number of markers did not always have a positive correlation. Indeed, Poland and collaborators [57], in wheat, achieved a lower accuracy on grain yield with 34,749 SNPs than Wimmer et al. [54] that analyzed the same CIMMYT wheat lines with only 2056 SNPs (~0.3 vs. ~0.47). Additionally, Xu [58] achieved a lower accuracy for flowering time in rice with 270,820 SNPs than Wimmer et al. [54], which used 36,901 SNPs (0.26 vs. 0.5). Windhaussen et al. [62] in maize obtained only a slightly higher accuracy for grain yield (~0.5) with 37,403 SNPs than Crossa et al. [27] with 1148 SNPs (0.42), although both authors used approximately the same TRN size and composition.

## 4. Tomato GS Schema Implementation

The establishment of GS experiment optimal parameters in a crop species requires a careful evaluation of key factors [71]. Plant selection response depends on the precision of the phenotyping and genotyping methods used to obtain the GEBVs (including the size of TRN, marker density, marker technology), knowledge of the genome structure, and marker linkage disequilibrium [26].

The success of modern breeding programs based on genomic techniques strictly depends on the precision of measurements related to phenotyped traits [72]. Digital instruments with scalable technologies can improve the precision of phenotyping [73], reduce the requirement of human data annotation, and accelerate the selection. Recent technologies have been used to acquire specific data on tomato traits with the aim of boosting the precision and the throughput of measurements, the size of analyzed plant populations, and, thus, enhancing the accuracy of the predicted phenotypic value and the genetic gain [74,75].

The appropriate TRN size and composition are also critical for gaining high prediction accuracy. A positive correlation between prediction accuracy and TRN size was confirmed in several species [76,77]. However, the optimal TRN size seems to be highly influenced by the relatedness of TRS and TST [25,78,79]. The highest prediction accuracies were found using TRS with a strong relationship to the TST [28,80,81]. Indeed, when the TRS and TST are unrelated, marker effects could be inconsistent due to the presence of different alleles, allele frequencies, and linkage phases. Developing ad hoc tomato TRN is crucial and update the TRN at each cycle could improve the prediction accuracy since the segregating population could accumulate genetic diversity and gene frequencies may change in each selection cycle [25].

To capture as many informative loci as possible an appropriate abundance of markers is required [82]. In this regard, genotyping-by-sequencing (GBS) can be used to efficiently generate high-density marker panels. Alternately, the cDNA-based GBS technique (RAR-seq restriction site-associated RNA sequencing) may detect conserved SNPs associated with a candidate mutation directly at the expression level [83]. Recently, a customizable method for tomato targeted genotyping, named single primer enrichment technology (SPET) was developed for improving the panel design and increasing the multiplexing levels of tomato genotyping [84]. Previous GS data can help to design an optimized suite of markers for the next steps. Liabeuf et al. [25] reduced the initial “SolCAP array” of 7700 SNPs [85] to screen populations with limited recombination. Moreover, the prediction accuracy may be also affected by the minor allele frequency threshold (MAF) [82]. Establishing methods for efficiently transferring validated genome signatures within tomato breeding selection procedures is also relevant. Human selection leads to changes in genomic regions that affect traits of agronomic interest, [86]. Detecting selection signatures is important for a better understanding of population history and genetic mechanisms affecting phenotypic cultivar differentiation [87]. Estimating allele or haplotype frequency differences between populations or generations within a population may improve traits selection. Linkage drag caused by recombination suppression can be reduced by estimating the effects of relevant markers improving prediction performance. Indeed, large gene introgression fragments in tomato cultivars from *Solanum* wild species caused drastic chromosome landscape changes. The *Solanum peruvianum* introgression carrying the tomato mosaic virus (ToMV) resistance gene *Tm2* can cover up to 79% of chromosome 9 in modern varieties [3]. To identify homologous recombination points, it was estimated that an average of 8× coverage should be used in tomato [88].

In the framework of GS, several statistical methods have been tested to estimate the marker effects in tomato [25]. The choice of the most appropriate method should be finalized to the specific context, considering the model complexity (genetic architecture, population size, and heritability) and the computation requirements [89,90]. Ridge regression best linear unbiased prediction (RR-BLUP) and genomic best linear unbiased prediction (G)BLUP [91], which assume a normal distribution of SNP effects, are suggested when assessing a trait that is affected by many small-effect genes using close TRN relatives. On the other hand, when traits are controlled by major-effect QTLs or when considering prediction of unrelated individuals, higher prediction accuracy can be obtained by Bayesian methods, considering a prior distribution of effects [92]. However empirical studies suggest that there are no major differences between regression-based and Bayesian methods in tomato [25].

## 5. Applying GS in Tomato Crop Improvement

Several constraints can affect the genetic gain of a GS program in the tomato. The implementation of GS requires the optimization of field trial management and agricultural practices, seed production, phenotyping, sample collection, and sequencing [93]. Moreover, as discussed above, parameters such as inbreeding level of populations, the number of individuals to be assessed, and marker metrics should be carefully evaluated to effectively run a GS-assisted breeding scheme. It can be estimated that, for tomato breeding programs, the genotyping work to complete GEBV predictions requires approximately three months. The selection decision will be achieved based on the higher GEBVs for each tested trait on the overall average of traits or as ‘indices’ of GEBV from several traits following selection priorities.

Once these issues have been addressed, the GEBVs can be calculated both to perform parental line selection and to evaluate the overall performance of the progenies in a descendent selection or backcross schemes.

The selection of elite parents to maximize the genetic variability exploitation is the first step in tomato F1 hybrid variety development. Elite germplasm represents a core collection of cross-compatible genotypes enriched for some favorable alleles [94]. Traditional breeding takes too much time in selecting elite lines. The main advantage of GS over traditional selection is that it can facilitate expeditious selection of superior variety/cultivar in less time by reducing breeding cycles [95].

In a GS-assisted breeding scheme for tomato F1 hybrid development, the decision to select parental lines is based on their breeding value (i.e., the mean performance of the progeny of a given parent) that consequently requires to be estimated accurately. Consistently, Yamamoto and collaborators [24] used a set of 96 big-fruited F1 tomato varieties to develop GS models, and the segregating populations obtained from crosses were used to validate the models. Consequently, the GS models were used to successfully predict parental combinations generating superior hybrids using progeny genotypic and phenotypic data for soluble solids content and total fruit weight. However, the efficiency of predictions varied depending on traits and parental combinations. While the need for fixing favorable alleles in the gene pool leads to increase inbreeding, the GS selection gain is dramatically reduced in small populations with narrow genetic variability. The managing of elite genetic diversity to increase the frequency of favorable alleles over time can highly benefit from GS approaches [94]. The prediction accuracy of parent cross ability could improve with the assessment of a higher number of selfing progenies. Thanks to the advances made in tomato genome knowledge and genotyping technologies, breeders can easily identify valuable alleles in elite germplasm [11,96] and create new lines combining these valuable alleles using a set of validated markers.

**Figure 2:**
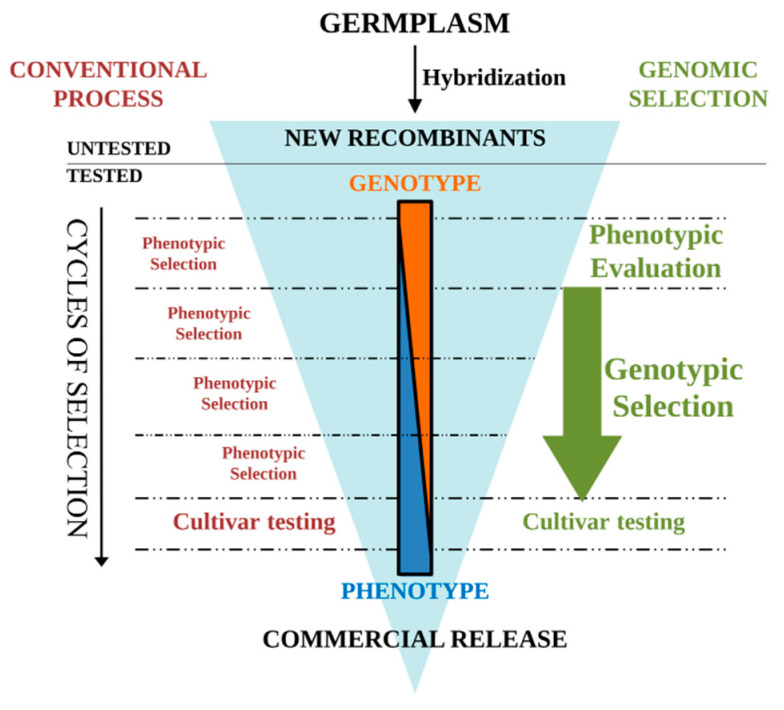
Comparison of genomic selection (GS) and conventional selection in tomato breeding programs. Screening of recombinant lines through GS approaches optimizes the genetic gain obtained in each selection cycle. Breeding cycles (horizontal dashed lines) are shortened by removing phenotypic evaluation of lines before training population (TRN) evaluation for the next cycle.

Generally, breeders take advantage of useful genetic variability by recycling the tomato best-performing varieties that have been successful for a given area by Single Seed Descendent (SSD) scheme where each generation derived from the former, taking only one seed from each parent plant. Nearly all steps can be conducted in the greenhouse, making this a method of choice for accelerating breeding in areas that do not benefit from a long enough growing season [97]. In the classical SSD scheme, the choice of tomato parental lines is very critical to ensure a higher additive breeding value since self-fertilization increases inbreeding level by 1/2 at each cycle. In the SSD scheme, no selection is conducted until the last generation (generally F6–F7), so the phenotyping of a larger number of lines could be challenging. The integration of the GS approach in the SSD could result in reducing the number of selfing generations thus shortening the overall selection process and decreasing the phenotyping effort (Figure 2). Because the prediction accuracy is generally higher when LD is high, an increase of the breeding gains is expected when applying GS in the earliest heterozygous segregating generations (i.e., F2–F4). Therefore, these generations could be successfully used for developing the GS model, and subsequently, GS prediction could assist selection in the following generations. Genomic data can accurately track the best performing plants along the generations, and the approach can successfully lead to the selection of individuals with the highest GEBV.

Backcrossing is another quite popular tomato breeding scheme employed to introgress a valuable trait from a donor parent into the genomic background of a recurrent parent. Backcrossing schemes with exotic or elite materials are widely used to introduce favorable traits. However, the constant introduction of novel alleles and the linkage drag, the crossing with old varieties or exotic material with low breeding value as well as the extended breeding cycles deriving from complex crossing scheme, can reduce the genetic gain per year. The response to genetic selection achieved through the selection of lines with high breeding value in a segregating population can be certainly improved by GS (Figure 2). A variant of the classical backcross scheme, where lines of each generation are selected based on recurrent parent breeding value, allowed researchers to obtain high rates of genetic gain [98,99]. By combining GS with single-marker assays, genes with major effects can be also selected within each offspring following the cross with the recurrent line. In this way, the GS approach is expected to additively increase the genetic gain at each generation. Candidate genotypes for selection, carrying specific alleles (i.e., resistance traits), can be identified using genotyping platforms that include gene-specific diagnostic markers or integrate single-locus data obtained with different technologies. In addition, among markers used in the GS model implementation, a subset of them identifying undesirable segments of a wild donor can be selected. In fact, large wild genome segments (between the 30 and 70% of the whole chromosome) were found to be incorporated due to resistance gene introgressions on a specific chromosome in cultivated tomatoes [3]. As an extension of this approach, genome-wide selection with high-throughput markers in BC1 could be even more efficient and the recovering of the recurrent parent genome could be increased from generation BC1 to BC3 without affecting favorable trait introgression.

## 6. Conclusions

The evaluation of complex traits such as disease resistance genes, QTLs for quality traits and abiotic environmental stresses (such as salinity, drought, and heat) with high efficiency in a segregating population can be a difficult task for tomato breeders. Innovative breeding strategies such as marker-assisted selection (MAS), high-throughput phenotyping, high-throughput genotyping, reverse breeding, and genomic selection, are now increasingly being used to complement the conventional approaches for the effective improvement of the tomato. In particular, the implementation of GS in breeding programs can accelerate genetic achievable gain if selection schemes will be tailored to genomic-guided procedures. This technique offers the possibility to double improve genetic gain. The acquisition of theoretical knowledge about tomato genome structure, evolution, and recombination can help to improve the practical application. The connection between genomic and phenotypic variations gives us the unique opportunity to predict bases on the genome and early in the life of individuals. We may not understand the underlying mechanism, but we can predict the results. Major GS implementation challenges were highlighted here, including model development, genotyping quality, optimal GS incorporation stage and indications for overcoming these issues were also discussed. While the methodological procedures begin to be delineated, the optimal way to incorporate GS in a breeding scheme remains to be empirically defined. Important features for the success of GS under different breeding scenarios should be assessed. Advancements in genotyping efficiency and phenotyping technologies will facilitate the adoption of GS in tomato breeding. A future update of existing selection schemes may be achieved using computer simulations for investigating different strategies to face the selection process gaps.

## Figures and Tables

**Table 1:** Genomic selection studies in plant species.

Species	Traits	TRN Size and Type	No Markers	Statistical Model	Accuracy	References
Wheat	GY	374 inbred lines	1158 DArTs	RR-BLIP, Bayes-A, B, C	0.21	[53]
Wheat	GY, HD	306 lines CIMMYT	1717 DArTs	RR-BLUP, Bayes-A, B, LASSO, RKHS, RBFNN, BRNN	0.7 0.5–0.6	[55]
Wheat	GY	254 lines CIMMYT	2056 SNPs	LASSO, Bayes-b, RR-BLUP	0.43–0.51	[54]
Wheat	GY	94 lines CIMMYT	234 DArTs	Bayes-LASSO-RKHS	0.43–0.79	[56]
Wheat	GY	254 lines CIMMYT	34,749 SNPs	GBLUP	0.2–0.4	[57]
Rice	FT	413 varietes	36,901 SNPs	LASSO, Bayes-b, RR-BLUP	~0.5	[54]
Rice	YP, FT, WSY	210 Inbred lines	270,820 SNPs	LASSO	0.16–0.26–0.98	[58]
*Arabidopsis*	FT	199 inbred lines	215,908 SNPs	RR-BLUP	0.65–0.75	[54]
*Arabidopsis*	FT, DM	415 RILs	69 SSRs	BLUP	0.90–0.93	[59]
Soybean	YP, PO	540 (RILs)	2647 SNPs	RR-BLUP	0.81, 0.71, 0.26	[60]
Soybean	nematode resistance	363 Genotypes	84,416 SNPs	RR-BLUP	0.41–0.52	[61]
Maize	GY, ASI	255 inbred lines	37,403 SNPs	RR-BLUP	~0.5	[62]
Maize	GY, FF, MF, ASI	300 lines CIMMYT	1148 SNPs	M-BL	0.42–0.79	[27]
Barley	GY, AA	150 DHs	223 RFLPs	BLUP	0.64–0.83	[59]
Barley	PH, CC	140 DHs	107 RFLPs, AFLPs	BLUP	0.66–0.85	[59]
Tomato	SSC, FW	96 F1 varietes	337 SNPs	GBLUP, Bayesian Lasso, Wbsr, BayesC, RKHS, RF	0.56–0.68 0.22–0.27	[24]
Tomato	Metabolic and quality traits	163 Genotypes	5995 SNPs	RR-BLUP	0.05–0.81	[22]

YP = yield; PO = protein oil; FT = flowering time; WSY = white sugar yield; DM = dry matter; GY = grain yield; ASI = anthesis-silking interval; FF = female flowering; MF = male flowering; AA = amylase activity; PH = plant height; CC = chemical components; SSC = soluble solid content; FW = fruit weight; HD = heading date.
